# Genetic Variant of DNAM-1 rs763361 C>T Is Associated with Ankylosing Spondylitis in a Mexican Population

**DOI:** 10.3390/cimb46040176

**Published:** 2024-03-23

**Authors:** Alejandro Vázquez-Reyes, José Francisco Zambrano-Zaragoza, Juan Manuel Agraz-Cibrián, Miriam Fabiola Ayón-Pérez, Gloria Yareli Gutiérrez-Silerio, Susana Del Toro-Arreola, Alan Guillermo Alejandre-González, Liliana Ortiz-Martínez, Jesse Haramati, Iris Celeste Tovar-Ocampo, Marcelo Victorio-De los Santos, Jorge Gutiérrez-Franco

**Affiliations:** 1 Unidad Académica de Ciencias Químico Biológicas y Farmacéuticas (UACQByF), Universidad Autónoma de Nayarit, Tepic 63000, Nayarit, Mexico; avazquez@uan.edu.mx (A.V.-R.);; 2 Laboratorio de Endocrinología y Nutrición, Departamento de Investigación Biomédica, Faculta de Medicina, Universidad Autónoma de Querétaro, Querétaro 76140, Querétaro, Mexico; 3 Instituto de Investigación en Enfermedades Crónico Degenerativas, Departamento de Biología Molecular y Genómica, Centro Universitario de Ciencias de la Salud (CUCS), Universidad de Guadalajara, Guadalajara 44340, Jalisco, Mexico; 4 Clínica de Reumatología, Servicio de Medicina Interna, Instituto Mexicano del Seguro Social (IMSS), Tepic 63000, Nayarit, Mexico; 5 Laboratorio de Inmunobiología, Departamento de Biología Celular y Molecular, Centro Universitario de Ciencias Biológicas y Agropecuarias (CUCBA), Universidad de Guadalajara, Guadalajara 44340, Jalisco, Mexico

**Keywords:** DNAM-1, ankylosing spondylitis, SNPs

## Abstract

DNAM-1 (CD226) is an activating receptor expressed in CD8+ T cells, NK cells, and monocytes. It has been reported that two SNPs in the *DNAM-1* gene, rs763361 C>T and rs727088 G>A, have been associated with different autoimmune diseases; however, the role of DNAM-1 in ankylosing spondylitis has been less studied. For this reason, we focused on the study of these two SNPs in association with ankylosing spondylitis. For this, 34 patients and 70 controls were analyzed using endpoint PCR with allele-specific primers. Our results suggest that rs763361 C>T is involved as a possible protective factor under the CT co-dominant model (OR = 0.34, 95% CI = 0.13–0.88, *p* = 0.022) and the CT + TT dominant model (OR = 0.39, 95% CI = 0.17–0.90, *p* = 0.025), while rs727088 G>A did not show an association with the disease in any of the inheritance models. When analyzing the relationships of the haplotypes, we found that the T + A haplotype (OR = 0.31, 95% CI = 0.13–0.73, *p* = 0.0083) is a protective factor for developing the disease. In conclusion, the CT and CT + TT variants of rs763361 C>T and the T + A haplotype were considered as protective factors for developing ankylosing spondylitis.

## 1. Introduction

Ankylosing spondylitis (AS) is a seronegative, inflammatory, autoimmune disease, which is considered the prototype of spondyloarthritis [1]. It is a disease with an important familiar genetic predisposition, and its genetic risk is estimated to be between 80 and 90% [2]. AS mainly affects young people and is more frequent in men than women, having a 2:1 ratio. The incidence and prevalence of AS depends on the region [3]. In Europe, the prevalence is 0.23%, it is 0.16% in Asia, and it is less common in Africa [4]. In Mexico, the prevalence of AS is estimated at 0.09% [5].

The main risk factor for developing the disease is the *HLA-B27* gene; however, other genes and molecules, especially those related to the activation of the immune system, could also be involved in the development of the disease [2]. To date, the association of genetic variations in numerous molecules with AS, such as endoplasmic reticulum aminopeptidase 1 (*ERAP-1*), interleukin 23 receptor (*IL-23R*), and interleukin 1 receptor 2 (*IL-1R2*), among others [1], has been reported. Additionally, a notable infiltrate of immune cells including macrophages, Natural Killers cells (NK), and T lymphocytes has been reported in the affected joints; these infiltrating cells can also play a role in bone damage through their activation at the site of the injury. Likewise, an increase in cytotoxic cells in circulation has been reported in patients with AS.

The DNAX Accessory Molecule receptor (DNAM-1) is a type 1, 65-kDa protein and is an activating receptor for CD8+ T lymphocytes (TCD8+) and NK cells [6]. DNAM-1 also functions as an adhesion molecule involved in the regulation of cytoxicity and the release of other cytokines by NK and CD8+ T cells [7]. Additionally, DNAM-1 is involved in communication with dendritic cells and functions as a bridge between innate and adaptive immunity [8].

Alterations in the expression of DNAM-1 have been reported in patients with cancer or human immunodeficiency virus (HIV), impacting the functionality of TCD8+ and NK cells [9]. The expression of DNAM-1 has been associated with cell maturation processes, especially in NK cells. This allows them to have different cytokine and chemokine expression profiles, favoring an inflammatory or regulatory state [10], as well as proliferation and functionality in TCD8+ cells [11].

The regulation of DNAM-1 expression has been suggested to be mediated by the transcriptional factor Eomesodermin (EOMES); however, EOMES is essential for the maturation and differentiation of TCD8+ and NK cells, so other factors could be involved in DNAM-1 expression [12]. This would indicate that other factors could alter the functionality of DNAM-1 and would play an important role in the function of TCD8+ cells and NK cells in different health and disease processes.

The role of DNAM-1 in AS is unknown, although there have been several reports associating single nucleotide polymorphisms (SNPs) in the *DNAM-1* gene with different autoimmune pathologies such as Type 1 diabetes mellitus (T1D), multiple sclerosis (MS), rheumatoid arthritis (RA), and systemic lupus erythematosus (SLE) [13,14,15,16]. In particular, two functional SNPs of the DNAM-1 receptor have been studied. The first is rs727088 G>A, which is found in the 3’-UTR region. This SNP has been associated with the low expression of messenger RNA and decreased protein levels in patients with SLE in NK, CD4+, and TCD8+ cells [17]. The second is rs767361 C>T, which induces the exchange of glycine at position 307 for serine. This change is believed to affect DNAM-1 signaling, as this substitution could affect three known phosphorylation sites at positions 319, 322, and 329 in the intracellular portion of DNAM-1 [14,18]. Serine 329 was previously reported to be crucial for DNAM-1 when interacting with LFA-1, allowing it to bind to lipid rafts during cell activation, allowing for the mobilization of calcium and the release of cytotoxic granules [19,20,21]. Therefore, both SNPs may play key roles in the activation of TCD8+ and NK cells.

Considering this information, the goal of the present study was to determine the association between *DNAM-1* and AS in a population from western Mexico.

## 2. Materials and Methods

We conducted a case–control pilot study. The study included a total of 34 patients diagnosed with AS (due to the low incidence of AS in the Mexican population), according to the modified New York criteria [22], at the Hospital de Zona No. 1, IMSS de Tepic, Nayarit, Mexico.

All samples were collected between 2012 and 2013, and all patients were undergoing treatment at the time of sample collection. A total of 70 healthy subjects with no family history of rheumatic or inflammatory diseases and no laboratory evidence of infections were included as the control group. All participants were Mexican residents from the State of Nayarit with ancestry of three generations from western Mexico, and signed an informed consent according to the Helsinki Declaration [23]. The blood samples from patients and controls were collected in EDTA tubes, and the genomic DNA was extracted by the modified Miller method. Samples were quantified using an Eppendorf Biophotometer^®^ D30 spectrophotometer (Eppendorf, Hamburg, Germany). The samples were stored at −80 °C until genotyping analysis.

The study was registered and approved by the Secretaría de Investigación y Posgrado (SIP) of the Autonomous University of Nayarit with the number SIP18-108.

### 2.1. Genotyping

**Figure 1:**
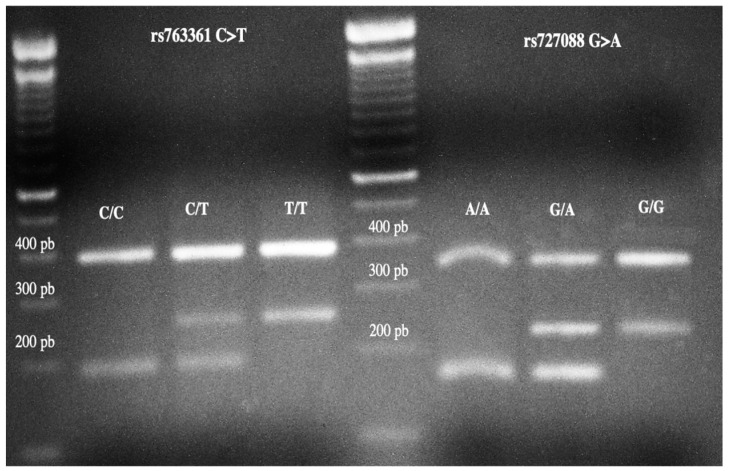
Representative image of the genotyping of SNPs rs763361 C>T and rs727088 G>A in 2% agarose gel. The size of the products for the SNP rs763361 C>T were 187 bp for allele C, 251 bp for alle T and 385 pb as internal control. For the SNP rs727088 G>A, product sizes were 161 bp for allele A, 235 bp for allele G, and 342 bp as internal control.

Genotyping of the rs763361 C>T and rs727088 G>A SNPs was performed by end-point PCR using oligonucleotides reported by Hashemi et al. [16]. Briefly, the concentrations used were as follows: 200 ng of genomic DNA in a total volume of 25 µL, containing 1X PCR buffer, 10 mM dNTPs, 15 mM MgCl2, 0.5U Taq DNA polymerase, and 10 pmol of each oligonucleotide for rs763361 C>T Forward Outer (FO): TTGCATAAAGATCCATGCATGAGTAC, Reverse outer (RO): GATTTCTGTTGCATCTCAGTCAAGAA, Forward inner (FI [T allele]): CATGGATTGATTGGTAGGTTGCCT, Reverse inner (RI [C allele]): CCAATAACTATAGAAGTCCCATCTCTAACG; and for rs727088 G>A Forward Outer (FO): TGTCATTAGGGCTGTCTTTGTCTGAATAG, Reverse outer (RO): CCAGGTCTAGCCTTAGGAGCAAATGTA, Forward inner (FI [G allele]): TTCCCTCCCAAATTTCTACCCTAACG, and Reverse inner (RI [A allele]): AGTGACAGTTGAAAGTGGTGGCATAGTAT. All reagents were from Invitrogen (Life Technologies Corporation, Carlsbad, CA, USA). The amplification was carried out in a Mastercycler gradient Eppendorf thermocycler (Eppendorf, Hamburg, Germany). The PCR conditions for the SNP rs763361 C>T were denaturation at 95 °C for 5 min, followed by 35 cycles of 95 °C for 30 s, 60 °C for 23 s, and 72 °C for 25 s, with a final extension of 72 °C for 10 min. For the SNP rs727088 G>A, the amplification started at 95 °C for 5 min and was followed by 35 cycles of 95 °C for 35 s, 64 °C for 35 s, and 72 °C for 26 s, with a final extension of 72 °C for 10 min. The PCR products were resolved on a 2% agarose gel stained with ethidium bromide (Figure 1).

### 2.2. Statistical Analyses

The allele, genotype frequencies, and haplotype analysis of both SNPs, along with the Hardy–Weinberg equilibrium, Odds Ratio (OR), and their respective 95% confidence intervals, were calculated using online SNPStats software (https://www.snpstats.net/, accessed on 10 November 2023) and the online WinEpi tool (http://www.winepi.net/sp/index.htm, accessed on 11 November 2023). Values of *p* < 0.05 were considered significant. In addition, the probability of multiple comparisons was adjusted using the Bonferroni correction method.

## 3. Results

In this work, we analyzed a total of 34 patients, and 70 controls were included. Table 1 shows the sociodemographic and clinical characteristics of the study groups.

We found that rs763361 C>T was in the Hardy–Weinberg equilibrium (patients *p* = 0.078, controls *p* = 0.81), while rs727088 G>A was not (patients *p* = 0.0003, controls *p* = 0.0001). The association between rs763361 C>T and rs727088 G>A in different genetic models is shown in Table 2. As can be seen, in rs763361 C>T, we observed a protective effect for the development of ankylosing spondylitis; this effect was seen in both the codominant model, CT variant (OR = 0.34, 95% CI = 0.13–0.88, *p* = 0.022), and in the dominant model, CT + TT (OR = 0.39, 95% CI = 0.17–0.90, *p* = 0.025). In contrast, the rs727088 G>A polymorphism was not found to have a significant indication as a risk factor for the development of AS using any of the different genetic models.

On the other hand, the haplotype analysis for both polymorphisms showed significant differences in the T + A haplotypes (OR = 0.31, 95% CI = 0.13–0.73, *p* = 0.0085) (Table 3), indicating that the combination of alleles T + A might protect against developing ankylosing spondylitis.

## 4. Discussion

DNAM-1 has been described as an important protein in immune function, and its involvement in various diseases has also been reported [24]. Considering this, our study focused on analyzing the association between the rs763361 C>T and rs727088 G>A polymorphisms of DNAM-1 with AS.

Our results indicated that the CT and CT + TT variants of rs763361 C>T were protective factors in the development of AS, while the SNP rs727088 G>A did not show any association in the different genetic models. Additionally, we found that the T + A haplotype could be a protective factor against developing AS.

Meta-analysis studies have shown that rs763361 C>T has been considered as a risk factor for autoimmune diseases in European, South American, and Asian populations, specifically with SLE, MS, T1D [25], and Wegener’s granulomatosis [26], but not in RA [25]; however, in the Iranian and Egyptian populations, it was considered as a risk factor for the development of RA [16,27]. Contrary to these reports, our results show that rs763361 C>T functions as a protective factor against AS. These discrepancies in our results are possibly related to the population studied or to the fact that some authors consider AS an inflammatory disease and not an autoimmune disease; this characterization is due to the activity of the innate immune system, critical interplay between mucosa, bacterial products, biomechanical stress and inflammation, and the marginal involvement of B and T cells—however, this is still up for debate [28].

In comparing the SNP rs727088 G>A with other autoimmune diseases, our results agree with the results obtained by Hashemi et al., who analyzed the SNP rs727088 G>A in patients with RA in the Iranian population and found no association with the disease [16]; however, Lofgren et al. studied a European population, and the SNP rs727088 G>A was reported to have a strong risk association in patients with SLE [17].

In the analysis of the haplotypes, we found that the T + A haplotype was a protective factor for developing AS; however, Löfgren et al. analyzed three SNPs of DNAM-1 (rs763361, rs34794968, and rs727088) and reported that the ATC haplotype was a risk factor for developing SLE, and that this was related to a decrease in the expression of DNAM-1 in CD4, CD8+, and NK T lymphocytes, but not in NK cells [17]. The variations in our results compared to other studies can be attributed to the different populations and numbers of samples analyzed.

We hypothesize that the effect of the CT variants found to be protective factors could influence the function of DNAM-1 in NK cells and CD8+ T lymphocytes, possibly inducing a sub-optimal activation state, thus reducing the inflammatory state. Nevertheless, more studies are needed to evaluate the function of CD8+ T lymphocytes and NK cells carrying each of the genetic variants. With our results, we can hypothesize that DNAM-1 could be involved in the pathophysiology of AS; however, future research is required to demonstrate the role of DNAM-1 in AS.

It is important to emphasize that our study is the first to investigate the association between genetic variants of DNAM-1, specifically the rs763361 and rs727088, with AS in the Mexican population. This study should be considered as a pilot as it is important to note that the study did not encompass sufficient patients for the rs727088 G>A to be in the Hardy–Weinberg equilibrium, and the age of the controls was not matched with the patient group. (We did not consider it necessary to age-match cases and controls, since our study focuses on evaluating only the protective or risk association with respect to the development of the disease, and AS is not a condition that is considered majorly dependent on age. However, in follow up studies, we plan to expand the patient and controls groups so that this assumption need not be made). In conclusion, this pilot study has allowed us to gather preliminary data and methodology that will facilitate the design of a larger-scale study with a greater number of patients, age-matched controls, and stratification of patients based on clinical and radiological characteristics. It is worth noting that our results should be interpreted with caution, and the aforementioned limitations should be taken into consideration.

## 5. Conclusions

In this study, we found that the CT and CT + TT variants of the SNP rs763361 C>T were a protective factor against AS, while the SNP rs727088 G>A was not associated with a susceptibility to the development of AS. Haplotype analysis revealed that the T + A haplotype acts as protective factor; however, these results should be considered preliminary, since this protective association was obtained in a low number of samples. Thus, it is necessary to increase the number of samples analyzed from this population.

## Figures and Tables

**Table 1:** Sociodemographic and clinical characteristics.

	Patients *n* = 34	Controls *n* = 70
Sex	30 Male	70 Male
	4 Female	0 Female
Age *	47.9 ± 13.02	20.75 ± 1.86
BASDAI * (0–10)	3.62 ± 1.92	ND
CRP * (mg/dL)	79.75 ± 58.11	ND
Treatment		
Sulfasalazine *n* (%)	31 (89)	-
Indometacine *n* (%)	22 (63)	-
Metrotexate *n* (%)	19 (54)	-
Prednisone *n* (%)	15 (43)	-
Others *n* (%)	30 (86)	-

ND: Not determined. Others: folic acid, diclofenac, acetaminophen, etanercept, adalimumab. * Mean ± SD.

**Table 2:** Association of DNAM-1 polymorphisms rs763361 and rs727088 in patients and controls.

	Patients	Controls	OR	*p*-Value
	*n* (%)	*n* (%)	(95% CI)	
rs763361				
Codominant				
CC	20 (59)	25 (36)	1.00	
CT	9 (27)	33 (47)	0.34 (0.13–0.88)	0.022 **
TT	5 (14)	12 (17)	0.52 (0.16–1.73)	0.281
Dominant				
CC	20 (59)	25 (36)	1.00	
CT + TT	14 (41)	45 (64)	0.39 (0.17–0.90)	0.025 *
Recessive				
CC + CT	29 (85)	58 (83)	1.00	0.752
TT	5 (15)	12 (17)	0.83 (0.27–2.59)	
Additive				
C	49	83	1	
T	19	57	0.56 (0.30–1.05)	0.072
rs727088				
Codominant				
AA	28 (82)	61 (87)	1.00	
GA	1 (3)	5 (7)	0.44 (0.05–3.91)	0.581
GG	5 (15)	4 (6)	2.72 (0.68–10.92)	0.144
Dominant				
AA	28 (82)	61 (87)	1.00	0.514
GA + GG	6 (18)	9 (13)	1.45 (0.47–4.48)	
Recessive				
AA + GA	29 (85)	66 (94)	1.00	
GG	5 (15)	4 (6)	2.84 (0.71–11.37)	0.126
Additive				
A	57	127	1.00	
G	11	13	1.885 (0.79–4.46)	0.144

* Statistical significance *p* ≤ 0.05; ** Bonferroni correction *p* = 0.025.

**Table 3:** Association between DNAM-1 haplotypes and risk of AS.

rs763361	rs727088	Freq.	OR (95% CI)	*p*-Value
C	A	0.6346	1	----
T	A	0.25	0.31 (0.13–0.73)	0.0085 **
T	G	0.1154	1.05 (0.53–2.09)	0.89
Global haplotypes associated *p* < 0.0071 *				
SNPs		D’	r	*p*-value
rs763361–rs727088		0.9994	0.4757	0.0

* Statistical significance *p* ≤ 0.05; ** Bonferroni correction *p* = 0.0125.

## Data Availability

Research data are available and stored in our immunology laboratory.
